# Health Sciences Students’ Attitude, Perception, and Experience of Using Educational Simulation in Saudi Arabia: A Cross-Sectional Study

**DOI:** 10.3390/nursrep12030061

**Published:** 2022-08-26

**Authors:** Ibrahim AlBalawi, Jaber S. Alqahtani, Shouq S. Al Ghamdi, Abdulelah M. Aldhahir, Musallam Alnasser, Abdullah S. Alqahtani, Saad M. AlRabeeah, Mohammed Alkhathami, Thekra N. Almaqati, Ibrahim A. AlDraiwiesh, Ameera K. Al Onezei, Arulanantham Zechariah Jebakumar, Yahya A. Alzahrani, Tope Oyelade, Eidan M. Alzahrani

**Affiliations:** 1 Advanced Clinical Simulation Center, Prince Sultan Military College of Health Sciences, Dammam 34313, Saudi Arabia; 2 Department of Respiratory Care, Prince Sultan Military College of Health Sciences, Dammam 34313, Saudi Arabia; 3 Anesthesia Technology Department, Prince Sultan Military College of Health Sciences, Dammam 34313, Saudi Arabia; 4 Respiratory Therapy Department, Faculty of Applied Medical Sciences, Jazan University, Jazan 45142, Saudi Arabia; 5 Clinical Laboratory Science Department, Prince Sultan Military College of Health Science, Dammam 34313, Saudi Arabia; 6 Nursing Department, Prince Sultan Military College of Health Science, Dammam 34313, Saudi Arabia; 7 Vice Deanship of Post Graduate Studies and Research, Prince Sultan Military College of Health Science, Dammam 34313, Saudi Arabia; 8 Respiratory Care Department, Imam Abdulrahman Bin Faisal University, Dammam 34212, Saudi Arabia; 9 Division of Medicine, University College London, London WC1E 6BT, UK; 10 Department of Physical Therapy, Prince Sultan Military College of Health Sciences, Dammam 34313, Saudi Arabia

**Keywords:** simulations, health sciences education, teaching, learning, higher education, nursing

## Abstract

*Background*: Simulation-based education (SBE) provides a safe, effective, and stimulating environment for training medical and healthcare students. This is especially valuable for skills that cannot be practiced on real patients due to ethical and practical reasons. We aimed to assess medical students’ attitude, perception, and experience of simulation-based medical education in Saudi Arabia. *Method*: A validated cross-sectional survey, using the KidSIM scale, was conducted to measure the level of perception and experience of students from different health sciences specialties toward integrating simulation as an educational tool. Participants responded to questions investigated the importance of simulation, opportunities for Inter-Professional Education (IPE), communication, roles and responsibilities, and situation awareness. Only students with previous experience of SBE were considered for participation. *Result*: This survey was completed by 246 participants, of whom 165 (67%) were male students and 228 (93%) were aged between the range of 18–30 years old. Of the respondents, 104 (67%) were respiratory care students, 90 (37%) were anesthesia technology students, and 45 (18%) were nursing students. Most of the participants had previous experience in IPE simulation activities (84%), and more than half of the students (54%) had a grade point average (GPA) ranging between 5.00 and 4.50. Overall, students had positive attitudes toward and beliefs about SBE, with a mean score of 129.76 ± 14.27, on the KidSIM scale, out of 150. Students’ GPA was significantly associated with a better perception to the relevance of simulation (*p* = 0.005), communication (*p* = 0.003), roles and responsibilities (*p* = 0.04), and situation awareness (*p* = 0.009). GPA is merely the sole predictor for positive attitude toward simulation with coefficient Beta value of 4.285 (*p* = 0.001). There were no significant correlations between other students’ characteristic variables (gender, specialty, study year, experience in IPE, and prior critical care experience). *Conclusion*: We found that health sciences students’ perception of SBE in Saudi Arabia is generally positive, and students’ performance is a significant determinant of the positive perception.

## 1. Introduction

As a means of mitigating risk during training in high-risk fields such as nuclear or aviation or medical fields, simulation is a viable teaching and learning tool [1]. Institutions and industries use it as a crucial part of training, education, certification, and ongoing license maintenance. Indeed, the value of simulation-based education has been demonstrated over the last century in a wide variety of industries and fields and has enjoyed great acceptance and diverse adaption by medical and health care educators [1,2].

Health care students and practitioners may benefit from simulation-based medical education because it is safe, engaging, and effective [3]. Simulation learning therefore represents a kind of experiential learning that is learner-centered, combines several components of learning (such as cognitive, motivational, emotional, psychomotor, and social), and offers a greater degree of realism [4]. Incorporating simulation into the classroom has the potential to be a very effective teaching strategy because it engages students, enables them to use critical thinking and clinical reasoning, and helps them to reflect on and integrate their prior knowledge [5]. The application of theoretical teaching technique to train students about how to manage patients is a major challenge for medical students [6]. On a global scale, the development of specialized clinical skills in disciplines such as pediatrics, emergency medicine, critical care medicine, obstetrics, anesthesia, radiology, and allied medical sciences has been facilitated through simulation [7,8]. Some medical institutions in the Middle East have updated their course offerings and used new teaching methods including problem-based learning and clinical skills-based lab education as part of their educational programs [9]. However, simulation-based learning using interprofessional education (IPE), is developing in this part of the world [10].

Indeed, simulation-based education (SBE) is gaining popularity as a method for teaching medical students essential psychomotor and critical thinking skills. This interest comes at a time when there is a growing need to teach these skills to ensure that a minimal management standard is assessed and attained prior to the beginning of patient care [11]. According to the World Health Organization (WHO), simulations may be helpful in education because students learn better in a safe, encouraging, stimulating, and interesting environment [12]. However, some medical students, especially those in advanced (trainee) level believed simulation-based clinical learning, did not accurately reflect clinical reality, which is often a hectic and an inconvenient setting [13]. Such unfavorable perceptions may impair the learning process by influencing student involvement in the SBE [14]. Therefore, to reap the benefits of the simulation, students must first comprehend the general principles so that they can sustain motivation and self-direction throughout the simulation experience [15]. Learners may do better than they would in everyday situations because they will likely behave more thoughtfully when they know they are being monitored [4]. There is a lot of concern that the emotional vulnerabilities of learners can be a critical challenge in simulation learning because of the high levels of emotional and motivational engagement that students typically describe [16]. As a result, it is crucial to investigate students’ attitudes, beliefs, and perceptions in order to provide them with an effective practice-based simulated learning environment [4].

In Saudi Arabia, where simulation is becoming more popular as an instructional tool in many medical sciences institutions [10,17,18], understanding students’ perceptions and attitudes toward various simulation methodologies is critical. Exploring undergraduate medical students’ attitudes and perceptions may reveal their preparedness to undertake simulations, since they affect their response, how they interpret scenarios, and how they act toward simulation. Therefore, this study aims to explore and evaluate health sciences students’ attitudes, perceptions, and experiences of using educational simulation in Saudi Arabia.

## 2. Materials and Methods

A validated survey was used to assess the medical students’ attitudes, perceptions, and experiences using educational simulation in Saudi Arabia [19]. We used a non-probability convenience sampling technique, because not all of the students have had simulation and also due to the availability of the students in the simulation center. The research team distributed the survey during the simulation training time for each specialty, in order to reach the target groups. Those who were absent were also approached in the following training session to reach more students. Inclusion criteria were all undergraduate students who had already received simulation-based training as part of their course study plan. Exclusion criteria were students with no prior experience of simulation, in addition to any students who did not agree to be a part of this study.

This study was conducted at Prince Sultan Military College of health Sciences, between March 2022 and June 2022 (Ref. IRB-2022-RC-028). We used a self-administered questionnaire for data collection using Google forms, which required 10 min to complete. The survey included two parts: the socio-demographic sheet and the KidSIM ATTITUDES questionnaire. The demographic part included self-reported gender, age in years, specialty, current study year, grade point average (GPA), past exposure to simulation, and other experiences. Our study adheres to STROBE reporting guidelines for observational research.

### 2.1. Study Survey Tool

The KidSIM survey was developed by Sigalet, Donnon, and Grant (2012) [18], and includes 30 items, with a high level of internal consistency reliability (Cronbach’s alpha = 0.95). The tool is a Likert-type scale with choices ranging from 1 to 5, with 1 being strongly disagree, and 5 being strongly agree, providing a range score between 30 and 150, with no items on the scale requiring reverse coding. The KidSIM survey questions capture the following: relevance of simulation, opportunities for IPE, communication, roles and responsibilities, and situation awareness (see [18] for details).

### 2.2. Power Calculation

The Prince Sultan Military College of Health Sciences includes 1300 students from several specializations, and the simulation center is solely used by four of them: Respiratory Care (RC), Anesthesia, Emergency medical services (EMS), and nursing programs. Thus, with a response distribution of 50%, a margin of error of 5%, with a confidence interval of 90%, and considering 1256 enrollments, the minimum sample size was 223 students.

### 2.3. Statistical Analysis

Students’ attitudes and perceptions regarding educational simulation were evaluated using descriptive statistics such as mean (M), standard deviation (SD), and percentages (%). *t*-test and ANOVA were used to assess the differences between groups. We also used multiple liner regression to assess the attitude score as dependent variable and find the predictors for positive attitudes considering all the student’s characteristics.

## 3. Results

### 3.1. Demographic Characteristics

This study includes 246 students who completed the questionnaire successfully. Table 1 presents the demographic characteristics of the study participants. The male participants comprised (67%, *n* = 165) of the total, while those between the ages of 18 and 30 years constituted a larger proportion of the sample (93%, *n* = 228). Most of the respondents were RC students (42%, *n* = 104), followed by anesthesia technology students (37%, *n* = 90), and nursing students (18%, *n* = 45). More than half of the participants (54%, *n* = 132) had a GPA between 5.00 and 4.50, and 84% (206) of them had participated previously in IPE simulation.

We asked respondents about previous simulated team learning, 121 (53%) of the respondents obtained their experience from their courses followed by seminars (26%, *n* = 92), workshops (24%, *n* = 84), and work experience (15%, *n* = 52). Furthermore, most respondents (*n* = 114, 46%) had no experience of critical care.

### 3.2. Attitudes, Perceptions, and Experiences Using Educational Simulation in Saudi Arabia

The overall students’ attitudes and perceptions about educational simulation were positive, with a mean (±SD) score of 129.76 ± 14.27 on the KidSIM scale, which has a range of 30 to 150. A total of 113 (46%) of the respondents had a score below the scale’s mean (129.76). Table 2 shows the details of the attitudes, perceptions, and experiences of the students in using simulation.

### 3.3. Student Characteristics Influencing Simulation Attitudes and Perceptions

ANOVA was used to examine the relationship between the students’ characteristics with regards to simulation survey elements. By comparing the mean result, significant differences were noted with respect to student GPA with regards to the statements related to relevance of simulation *p* = 0.005, communication *p* = 0.003, roles and responsibilities *p* = 0.04, and situation awareness *p* = 0.009. In addition, significant differences were found with respect to age group with the statements related to roles and responsibilities *p* = 0.02. However, no significant difference was found between other variables (gender, specialty, study year, IPE simulation, and previous critical care experience) in relation to the simulation survey response. Table 3 shows the mean of the students’ scores on the five subscales of the KidSIM questionnaire.

### 3.4. Predictors of Positive Students’ Attitudes and Perception toward Simulation

As shown in Table 4, multiple linear regression analysis was made to identify the significant predictor of the students’ total score among the independent variables such as gender, specialty, current year of study, GPA, previous experience on IPE simulation, and Critical care experience. The resulting model was significant (F = 3.15; *p* < 0.05) with no effect of gender, specialty, study year, IPE simulation, or previous critical care experience. Only GPA was significantly related with coefficient Beta value of 4.285, which indicates higher GPA result led to better attitudes about educational simulation.

## 4. Discussion

We present herein the general attitude, perception, and experience of 246 medical students of simulation-based learning in the Kingdom of Saudi Arabia. The main findings of this study are that medical students generally perceive simulation-based learning as a useful tool that offers a good learning environment and fosters teamwork, improve professional communication, helps student to understand their roles and responsibility and improves situation awareness. We also found that students GPA is the main driving factor for their perception of usefulness of simulation-based learning whereby high-performing students find the simulations more useful.

The use of simulation in medical training provides a safe, effective, ethical, and cost-effective learning environment which can be modified for a variety of medical specialties [7,15,20]. Simulated team training may also be used to develop relevant skills needed in real life clinical environments including leadership, communication, and decision-making skills. In surgical training, simulation-based learning technology is a great tool for surgeons to horn their skills in a relatively risk-free environment where tutors’ feedbacks could be provided in real time [21,22,23].

Our result herein corroborates a previous report by Joseph et al. [24] involving 247 medical students from the south of India where perception of simulation-based medical training was positively perceived and purported as a bright prospect which may improve learning. Similar findings have also been reported in a survey of third year medical students and their tutors that used mime-based role-playing as a form of simulation-based learning of neurological semiology in France. The authors found that more than 70% of students perceived that the technique increased their learning motivation, subject understanding, and knowledge retention and believed that the simulations would improve their clinical practice in future [25].

Furthermore, both tutors and students also encouraged the inclusion of simulation-based learning techniques in medical education [25]. Indeed, in another study on tutors’ perception of simulation-based training, authors reported a generally positive outlook with few passive barriers including provision of adequate training support and learning/teaching resources [26]. In addition, because of their ability to explicitly customize standardized circumstances to test performance, simulations serve effectively concerns of quality control, certification, and re-certification. Simulations provide a high degree of standardization in the evaluation of diverse competencies and practices, and are therefore viewed as fairer by students than unstandardized evaluations [4].

While the use of simulation-based techniques has become the norm and an integral part of healthcare education, the perceived advantages cut across various other learning fields as well. For instance, in a survey of 114 second year engineering students from Singapore, students reported that simulation-based learning meets their psychological needs and may improve motivation and learning outcomes [27]. This was also reported by Hunziker et al., where students often express high levels of emotional and motivational engagement associated with simulation learning [16].

Put together, our study corroborates previous study to provide further prove that simulation-based education is positively perceived by students and is a viable tool in healthcare and medical education. Furthermore, we provide the first evidence that high-performing students are relatively more likely to have positive perception of simulation-based learning. Therefore, the adoption of SBE in Saudi Arabia is a critical milestone in curriculum planning. When it comes to medical education, SBE may help in several ways, including lowering risks for both students and patients, boosting students’ competencies and self-assurance, enhancing care for patients, and decreasing overall healthcare expenditures.

A main limitation of this study is that most participants are high-performing with more than 50% having a first-class GPA (4.50–5.00), although this is expected of medical and healthcare students. Indeed, because high-performing students are likely to have positive perception, this may be a source of bias. However, the sampling method is completely random, and the authors have no power to affect the performance of students that responded to the survey. Second, the population of participants was drawn from a single institution with strong history of curricular simulation-based learning, thus the findings may not represent the perception of other institutions relatively less engaged in simulation-based education. While focus groups were shown to be useful for studying attitudes and beliefs, we did not include them in our research. Indeed, future studies should include more topically diverse students with various levels of performance to bring to light the inter-disciplinary perception of simulation-based learning by students. This could help increase awareness and integration for general learning and improve students’ learning as well as the tutors’ teaching experience.

## 5. Conclusions

The study’s key results include that health sciences students regard SBE as a beneficial tool that develops teamwork, enhances professional communication, helps students understand their roles and responsibilities, and increases situation awareness. We also observed that students’ GPA is the primary determinant of their perception of the value of simulation-based learning, with high-performing students finding the simulations more beneficial. Consequently, SBE incorporation into Saudi Arabia’s educational system should be taken into account throughout the curriculum’s designing phase.

## Figures and Tables

**Table 1:** Demographics and background characteristics of the students (*N* = 246).

Variable	*N* (%)
**Gender**	
Male	165 (67%)
Female	81 (33%)
**Age Range**	
18–30	228 (93%)
31–40	16 (7%)
41–50	2 (1%)
**Specialty**	
RC	104 (42%)
EMS	7 (3%)
ANES	90 (37%)
Nursing	45 (18%)
**Current Study year**	
Second year	70 (28%)
Third year	79 (32%)
Fourth year	60 (24%)
Internship	37 (15%)
**GPA**	
5.00–4.50	132 (54%)
4.49–3.75	94 (38%)
3.74–2.75	15 (6%)
2.74–2.00	3 (1%)
Less than 2.00	2 (1%)
**Have you participated in Inter-professional Education (IPE) simulation previously?**	
Yes	206 (84%)
No	40 (16%)
**Previous Team Based Learning**	
Workshop	84 (24%)
Seminar	92 (26%)
Course	121 (35%)
Work Experience	52 (15%)
**Critical Care Experience**	
None	114 (46%)
<1 week	49 (20%)
<2 week	12 (5%)
<3 week	17 (7%)
1 month	11 (4%)
>1 month	43 (17%)

**Table 2:** Attitudes, perceptions, and experiences using educational simulation in Saudi Arabia.

Item	Mean ± SD
**Relevance of Simulation**	
1. Simulation is a good environment for learning with other health care professionals	**4.48 ± 0.692**
2. Simulation supports opportunities to change attitudes	**4.39 ± 0.701**
3. Opportunities to practice teamwork can help students learn about inter-professional roles	**4.32 ± 0.692**
4. Opportunities to learn with other health care professionals has increased my understanding of their roles	**4.34 ± 0.703**
5. Simulation is a good tool for practicing team decision-making skills	**4.25 ± 0.762**
6. Deliberate practice can improve clinical decision-making skills	**4.23 ± 0.775**
**Opportunities for Interprofessional Education (IPE)**	
7. Learning with other professionals is important to collaboration	**4.30 ± 0.733**
8. Opportunities to learn with other professionals should be a priority in my education	**4.16 ± 0.817**
9. I want more opportunities to learn with other professionals	**4.28 ± 0.831**
10. Shared learning with other team members will improve my ability to understand clinical problems	**4.40 ± 0.655**
11. Attitudes about teamwork can change through opportunities to work with other professionals in simulation	**4.21 ± 0.742**
12. Learning with other health care professionals before qualification is important for the development of future inter-professional relationships	**4.28 ± 0.775**
13. Interprofessional opportunities for learning will improve patient outcomes	**4.32 ± 0.704**
**Communication**	
14. All students should learn how to work in the context of health care teams	**4.32 ± 0.738**
15. Team leaders should provide frequent patient updates to other team members	**4.35 ± 0.705**
16. Team leaders should encourage team members to ask questions	**4.37 ± 0.731**
17. Communication within the team is as important as technical skills	**4.46 ± 0.703**
18. Team members providing immediate patient care management should verbalize their activities aloud	**4.20 ± 0.805**
19. Team members should paraphrase or repeat back instructions to clarify their understanding	**4.17 ± 0.785**
20. Communication in teamwork is important to patient safety	**4.44 ± 0.730**
21. The roles of non-leading members of the team are just as important for good team functioning as the role of the leader	**4.29 ± 0.789**
**Roles and Responsibilities**	
22. Teamwork practice will provide me with feedback to enhance my ability to provide optimal patient care	**4.39 ± 0.712**
23. Monitoring what each team member is doing is important to optimize patient safety	**4.35 ± 0.739**
24. Will enhance other team members understanding of my role in patient health care	**4.28 ± 0.723**
25. Teamwork practice will help me recognize how best to help other team members complete their tasks	**4.32 ± 0.744**
26. It is important for team members to ask for assistance if they need support in completing a task	**4.38 ± 0.740**
27. Teamwork practice allows for flexibility in roles during times of crisis	**4.36 ± 0.774**
**Situation Awareness**	
28. I will speak up if I perceive a problem regardless of who might be affected	**4.30 ± 0.783**
29. Patient care is improved when all team members have a shared understanding of the assessment and treatment	**4.43 ± 0.701**
30. Team leaders should provide frequent summaries of patient findings to keep team members oriented to patient needs	**4.41 ± 0.721**
**Total Score (30–150)**	**129.76 (14.27)**

BOLD indicates the main domains for the survey.

**Table 3:** The mean total of the students’ scores on the five subscales of the KidSIM questionnaire.

Type of the Items	Number of Items	Sub Scale Potential Range	Mean (SD)
Relevance of Simulation	6	6–30	26.00 (3.22)
Opportunities for Inter professional Education (IPE)	7	7–35	29.95 (3.83)
Communication	8	8–40	34.59 (4.36)
Roles and Responsibilities	6	6–30	26.08 (3.50)
Situation Awareness	3	3–15	13.14 (1.71)
Total	30	30–150	129.76 (14.27)

**Table 4:** Predictors of positive students’ attitudes and perception toward simulation.

Variables	Beta (95%CI)	SEM	*p*-Value
Constant	113.418 (98.314–128.523)	7.668	0.001
Gender	−4.453 (−9.365–0.458)	2.493	0.075
Specialty	−0.605 (−2.795–1.585)	1.112	0.587
Current study year	1.756 (−0.07–3.582)	0.927	0.059
GPA	4.285 (1.828–6.741)	1.247	0.001
Experience in Interprofessional Education simulation	−0.26 (−5.066–4.546)	2.44	0.915
Critical Care Experience	−1.066 (−2.247–0.115)	0.6	0.077

## Data Availability

The data presented in this study are available on reasonable request from the corresponding author.
